# Lived experiences of Malaysian family caregivers of patients with chronic illnesses: A qualitative study

**DOI:** 10.51866/oa.683

**Published:** 2025-05-08

**Authors:** Nurul Salwa Sajali, Norfaezah Md Khalid, Norsafatul Aznin A Razak

**Affiliations:** 1 BCouns, PhD Couns, Department of Educational Psychology and Counseling, University of Malaya, Kuala Lumpur, Malaysia. Email: norfaezah@um.edu.my; 2 B.H.S (English Lang. & Lit.), MSSc (Couns Psych), Department of Educational Psychology and Counseling, University of Malaya, Kuala Lumpur, Malaysia.; 3 BCouns, PhD Couns, Department of Educational Psychology and Counseling, University of Malaya, Kuala Lumpur, Malaysia.

**Keywords:** Understanding, Family caregivers, Chronic illness, Qualitative research

## Abstract

**Introduction:**

Chronic illnesses have added to the demand in informal caregiving. Culturally, family members are expected to provide physical care and psychosocial support to their sick members, but the challenges they face have adversely impacted their employment, financial, physical and emotional wellbeing. This study aimed to explore the lived experiences of family caregivers of patients with chronic illnesses.

**Methods:**

This qualitative research was based on the interpretative phenomenological analysis (IPA) approach. Purposive sampling was used to recruit five family caregivers of patients with chronic illnesses. Data were collected through in-depth semi-structured individual interviews and document analysis and later analysed systematically according to the six-step IPA process.

**Results:**

The data revealed the informants’ meaning of experience in four themes: 1) adversities, 2) adjustment challenges, 3) adaptation and acceptance and 4) identity.

**Conclusion:**

The findings suggest that constructive lived experiences may be cultivated by increasing knowledge in caregiving, receiving greater appreciation and support from family members and learning to embrace the new identity. Caregiver support programmes or workshops focusing on emotional resilience or communication strategies can help to boost the positive life experiences of caregivers. The findings can be useful for healthcare professionals in understanding and addressing the needs of caregivers and improving the support and services provided to them.

## Introduction

A chronic illness is a condition that endures for a long time, which includes major diseases and conditions such as heart diseases, cancer, diabetes and arthritis.^1^ Major chronic conditions are long-lasting, slow-progressing and not contagious; they are divided into four main types: cardiovascular diseases (e.g. heart attacks and stroke), cancers, chronic respiratory diseases (e.g. chronic obstructive pulmonary disease and asthma) and diabetes.^2^ Chronic illnesses typically last for 3 months or more and generally cannot be cured by medication or prevented by vaccines.^3^ The variation in the terminology for chronic illnesses is apparent, but it can be concluded that having a chronic illness requires ongoing medical care and consistently limits a person’s daily activities, which can be heartbreaking, painstaking and challenging. Life with a chronic illness typically requires ongoing changes that may be demanding or stressful for both patients and their families. In 2019, 16% of informal caregivers in Malaysia reported that caregiving deteriorated their health physically and/or mentally.^4^ In most situations, patient caregivers must juggle multiple responsibilities simultaneously, leading them to experience various forms of stress - especially when caregiving duties are carried out without the help and support of immediate family members. In worse cases, suicidal ideation and death by suicide have been reported among caregivers, including people caring for family members with dementia, schizophrenia and cancer.^5-8^ Ultimately, the response to caregiving depends not only on the nature and course of the illness but also on the family itself. Family roles and history influence the health and illness of a child’s chronic illness trajectory.^9^ Consequently, the complementarity between family members during caregiving takes the form of teamwork; without this, the family will fail to serve its purpose as a support system for one another.

In the Malaysian context, family caregiving is often seen as an obligation, and this understanding has been passed down through generations.^10^ Additionally, family functioning practices are collectivist, where interdependence, family embeddedness and connectedness and relationship hierarchies are embraced.^11^ Nevertheless, the nonconfrontational approach, which values respect and politeness based on hierarchical relationships, often backfires. In caregiving, caregivers may view their duties as part of their responsibilities and may be reluctant to acknowledge the burden they carry. However, when caregivers are pushed beyond their limits and protective measures are no longer effective, the burden can manifest and lead to stress.^12^

Family caregivers in Malaysia may differ from those in other countries considering the differences in the healthcare system, culture, socio-economic status and educational background.^13^ In reality, many are unwilling to share details about their family life in the name of honour and unity, to the extent that help-seeking behaviour is hindered. In Malaysia, important knowledge gaps persist, particularly regarding the complexity and diversity of family dynamics.^14^ Therefore, the present qualitative research aimed to explore the lived experiences of family caregivers and enrich the existing caregiving literature in the Malaysian context.

## Methods

### Research design

This study employed a qualitative research design, specifically the interpretative phenomenological analysis (IPA) approach, aiming to explore family functioning from the perspectives of family caregivers. The main difference of IPA from other qualitative approaches is the interpretative and systematic process between researchers and the researched, with a focus on how a specific phenomenon has been perceived from the perspective of certain individuals in a particular and complex c ontext.^15^

### Sampling

Purposive sampling is a criterion-based selection technique that guides researchers in creating a list of informant criteria fundamental to their study.^16^ In this study, the inclusion criteria were an age of >18 years, residence in the same household as the patient with a chronic illness and a caregiving duration of >6 months. In IPA, the recommended number of informants is three to six; however, for doctoral studies, it is recommended to focus on the number of interviews (six to ten), as the objective is to produce quality data.^15^ Hence, in this study, five informants were included, each participating in a three-series interview; they represented the phenomenon.

### Procedures and data collection methods

Data were collected through in-depth interviews and reflexive journaling. A previously developed interview protocol was reviewed by three field experts: a qualitative research expert, a family therapy expert and a counselling expert. However, the protocol served as a basic guideline and was open for development based on informants’ information. Both face-to-face and online semi-structured interviews were conducted by one researcher. Each interview lasted for 1–1.5 hours and was recorded on an MP3 player and videotaped. Each informant was interviewed three times following the three-series interview guidelines by Seidman.^17^ The first series focused on rapport, the second series on informants’ history of caregiving and the third series on their trajectory as a caregiver. All interviews were completed within 3 months.

### Data analysis

Data were analysed following the step-by-step IPA process as summarised in Figurel.

**Figure 1:**
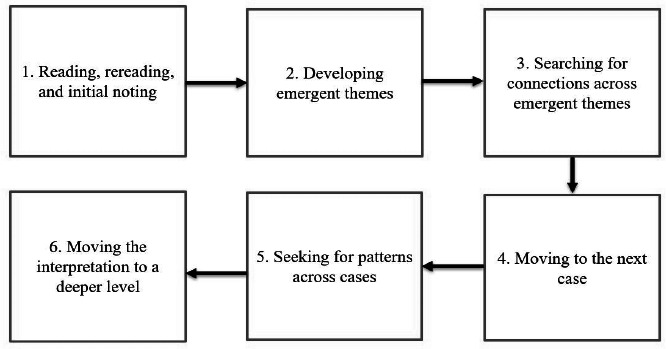
Six steps of the interpretative phenomenological analysis approach.

### Steps

In the first step, free textual analysis was conducted to achieve familiarity with the overall experience and assist in the theme development. Coding, including labelling and organising data through line-by-line inspection to identify emergent themes that answer the research questions, was also performed.

In the second step, the researchers identified emergent themes by breaking the entire transcript into discrete chunks. At this stage, the data reading became more intensive and interpretative. The researchers analysed the interview transcripts and reflexive journal to understand the meaning-making significance for each informant. The emerging themes were both expressive and concise, addressing the central objectives of this study.

In the third step, the researchers arranged the themes in chronological order and moved them to form clusters of related themes. Related themes were grouped together, while nonrelated themes were placed opposite each other in the document. During this stage, some themes were discarded. This step was repeated and involved multiple attempts of rearranging the themes according to the research questions. Abstraction was used to group the themes together, placing related themes together (subordinate themes) and developing a new name for the cluster (superordinate themes).

The fourth step involved repeating the previous steps for the other transcripts of informants. This was essential to treat each transcript on its own terms, without relating it to the previous one. This process was also considered bracketing, ensuring the researchers remained objective while analysing the data. Subsequently, the researchers identified patterns across cases. At this stage, fixed superordinate themes were developed while still maintaining a focus on individual particularity.

Finally, the researchers deepened the interpretation by reconfiguring and relabelling some of the themes using theories and models to frame the analysis. At this stage, they referred to established family functioning models and theories such as the McMaster model of family functioning^18^ and the structural family theory.^19^ The findings were then illustrated with relevant quotes from informants, accompanied by detailed interpretative commentary. This study also used NVivo 12 to organise, manage and code multiple data sources in one central file.

### Document analysis

Bracketing (also known as epoche) is the process of setting aside one’s personal experiences, biases and assumptions to adopt a fresh perspective of the phenomenon of interest.^15^ Reflexive journaling is a common practice for researchers while engaging in bracketing throughout the research process. In this study, the researchers recorded their personal experiences, values, beliefs and interests during the research. They also included informants’ information and their nonverbal behaviours from their perspectives at the end of each interview. This ensured that all researchers’ observation and reflection would not be distorted by time and influenced by other informants’ interview sessions.

### Data trustworthiness

Data triangulation was conducted: The researchers compared and cross-checked the data collected from the interviews and reflexive journaling. Member-checking was also performed, wherein the researchers summarised their understanding of the data and verified them with informants. All informants agreed with the early findings shared to them. Additionally, peer review, involving discussions with experts and co-researchers, was conducted to reduce researcher bias. During peer review sessions, thorough discussions of the emerging findings took place to reach a final consensus. The researchers’ reflexive journal served as a tool for reflexivity and an audit trail, documenting the study’s process, methods, procedures and decisions. Furthermore, the transferability of the data was ensured through the diversity in purposive sampling, data collection method, data analysis and the replication of research procedures for other researchers.

## Results

A total of five Malay-Muslim informants caring for their parents were interviewed in this study. Among them, two were men, while the other three were women. The profile of the informants is shown in Table 1.

**Table 1:** Informant profile.

Informant	Age (year)	Sex	Occupation	Household income	Marital status	Patient’s chronic illness	Period of caregiving (year)	Relationship with patient
Marigold	28	Male	Business owner	MYR 5000-10,000	Married	Kidney failure, heart disease	6	Father-son
Allium	38	Male	Teacher	MYR 5000-10,000	Married	Kidney failure	6	Mother-daughter
Rose	52	Female	Housewife	MYR 5000-10,000	Divorced	Bone marrow cancer	6	Father-daughter
Peony	40	Female	Unemployed	MYR 5000-10,000	Married	Stroke	3	Mother-daughter
Tulip	26	Female	Teacher	MYR 5000-10,000	Married	Breast cancer	4	Mother-daughter

Table 2 shows the final themes and subthemes of the study. Four themes emerged from the data collected and analysed from the five informants. These themes included 1) adversities, 2) adjustment challenges, 3) adaptation and acceptance and 4) identity.

**Table 2:** Themes, subthemes and representative quotes.

Theme	Subtheme	Representative quote
Theme 1: Adversities	Limitation in preparation, knowledge and skills	*So, I personally think caring for my father is a very tough process. Even my other family members always complain about my father during the beginning of caregiving. So, it’s very stressful.* (Marigold) *Undeniably, there was no emotional preparation. Mentally and physically, everything was chaotic at the time. During the day, I work as a teacher, and at night, I must stay awake to care for my mother.* (Allium) *I was surprised because all of a sudden, she was no longer healthy; she was like a baby again. In the early days, I struggled a lot. Everything was new to me; even the simplest task like putting on or changing diapers was stressful.* (Peony)
	Career is secondary	*When I was at work, my mother would call and ask me to go back. I had no choice but to leave her alone due to work. It was a constant pressure, but she is my priority and responsibility, so I would leave my work and go back home.* (Allium) *I used to take emergency leave so often because my siblings were too busy with their own commitments, which eventually caused me to be laid off. I was neither sad nor angry because all I could think of at that time was who will take care of my mother.* (Peony) *I had to apply for leaves for months. I received lots of warning letters before that, as I always took emergency leaves. My colleagues and school were understanding, but that’s the procedure.* (Tulip)
	Psychological distress	*Sometimes, I am emotionally disturbed. I cannot sleep and feel exhausted at all times. So, no doubt, I had my outbursts, too.* (Allium) *I always keep my frustrations at heart, as I don’t want to make things worse, but the effect would be constant headaches and fatigue.* (Rose) *I am not an outspoken person. So whenever I disagree with others, I would just keep quiet and distance myself. I often cried and was stressed out after that.* (Peony)
Theme 2: Adjustment challenges	Role ambiguity	*When taking care of my mother, it was like I had to forget my other roles and responsibilities. I am fortunate to have an understanding wife; she bears all the responsibility at home, taking care of our twins and working at the same time.* (Allium) *Yes, caring for my father is my top priority, but it is impossible to have balance. One of my kids is autistic, so yeah, it’s hard to be a daughter and a mother at the same time.* (Rose) *I value my personal life and freedom. Yet, when my mother got sick, it was like a turning point. I struggle to meet my own needs, but it’s not really a dilemma, as I am willing to sacrifice anything for my mother.* (Tulip)
	Lack of unity	*They are selfish. I have kids, too; I have a wife, too; I have a family, too. I struggle with caregiving, but they are happily enjoying life with their family. They don’t even visit my mother during their free time.* (Allium) *I’m so tired, you know. I’m so tired, as if the other siblings don’t exist. Why? Why does it have to be me alone? Such is the thought. Yes, I lived here with my parents, but that doesn’t mean everything is my responsibility.* (Rose) *My relationship with my siblings was good before caregiving. When my mother fell sick, everything changed 100%. None of them were willing to take care of my mother; they showed their true colours.* (Peony)
	Family conflict	*Communication in my family is worst especially in solving issues or conflict or cooperating with each other. So, indeed, the main reason is that our relationship had already been strained long before caregiving. I find it hard to feel a connection with them.* (Marigold) *My second eldest brother is rarely involved in family matters. My sisters are all married, but I am also married and have my own family. They always argue about who should take which caregiving shifts.* (Allium) *My father is a difficult person. When my mother fell sick, he was always complaining, nagging and refusing to take part in caregiving. He always told us, the children, who must take care of my mother. I was tired of my other siblings and his temperament, so when my youngest sister suggested bringing my mother to her place, I immediately agreed.* (Peony)
Theme 3: Adaptation and acceptance	Filial piety	*So, I always thought I couldn’t do much, but now I have no choice because I bear the responsibility as the only son.* (Marigold) *But when the time comes... its as if its instinctive that we, as the children, have to take care of them. It’s our time to repay their kindness and love.* (Allium) *It’s like karma. What you give, you get back. One day, I will also be sick. If God wills, my kids will take care of me the way I take care of my father.* (Rose)
	Strengthened bonds	*Somehow, when all were at home, and my father was sleeping, we were spending time together without realising it. We would order fast food and watch movies for a few hours.* (Marigold) *I try to express affection while taking care of him. In that sense, our relationship has become closer compared to before, as we rarely had that kind of physical touch. So, when he’s sick, the physical touch increases because I feed him, wipe him, brush his hair and stay with him all the time.* (Rose) *My relationship with my mother is much better now, and she is more cooperative. She can’t talk, but I know she appreciates my sacrifices.* (Peony) *I get to spend time with her; we always share stories, and we’ve become closer than before. The noblest thing about my mother is she always puts me first. ‘Have you eaten? Do you need money?’ Being sick hasnt changed her even a bit.* (Tulip)
	Religious belief	*For me, religion is very important; it is the source of strength for all of us.* (Marigold) *I put my trust in Allah. Yukallifullahu nafsan illa wus’aha. God will not impose something on a person unless Allah knows that he is capable of doing it.* (Allium) *I always believed in Allah’s plan. This is His way to make me a better person.* (Peony)
Theme 4: Identity	Loss of self	*So, what happened was I naturally cut off everything; I no longer have a social life. My life is all about working, returning home, caregiving and taking care of the family.* (Marigold) *I always feel guilty towards my wife. I may be a good son but not a good husband and father.* (Allium) *I don’t have time for myself or friends. My life now is going to work, returning home, taking care of my mother and falling asleep due to fatigue.* (Tulip)
	Caregiver traits	*Despite all, I am more resilient and sincere and focus on my best. My brother was my role model, and I will not let his sacrifices be in vain.* (Marigold) *Personally, I am more understanding and resilient. I am also a better listener now.* (Allium) *My caregiving journey has humbled me in many ways. As a person, I am more emphatic, forgiving and resilient.* (Rose) *So, I think my empathy has grown, and I am more resilient. I always remind myself that no one will be healthy forever.* (Tulip)
	Emotional maturity	*I no longer have high hopes or expectations. My main goal is to have no regret if one day my father dies.* (Marigold) *I am focusing on providing comfort and happiness for my mother – my main priority. I have learnt that things that are out of my control are not my concerns.* (Allium) *I have become selfless and really wish the best for my mother. I only hope she is comfortable and enjoys every moment. These are her moments, so I will make sure she is in good hands.* (Peony)

### Theme 1: Adversities

The adversities reported reflected the informants’ struggles and feelings, impacting different aspects of their lives. At the early stage of caregiving, the informants described the situation as poorly received and overwhelming due to limited preparation, knowledge and skills. Taking care of sick family members also left them with no choice but to make sacrifices. One informant was terminated from their job, while another had to prioritise caregiving over their career to better understand and tend to the needs of the sick. Eventually, this led to psychological distress, as they perceived caregiving as both physically demanding and highly stressful.

### Theme 2: Adjustment challenges

Despite all the adversities, the informants provided their full commitment and time to their care recipients. They also recognised that the main obstacles in the adjustment process and the integration of caregiving into their daily lives were the lack of support and family conflict. The absence of support from people around them made it difficult for them to carry out their duties well at home. This was because they had to juggle multiple roles simultaneously, yet others, especially family members, refused to participate or provide the necessary help. The informants further highlighted that the difficulty of the adjustment period was exacerbated by the constant rejection of involvement by family members due to existing family conflicts, either between care recipients and family members or between caregivers and other family members. In some cases, caregiving created more conflict within the family system rather than fostering harmony.

### Theme 3: Adaptation and acceptance

The long-term caregiving experience actually equipped the informants to adapt to and accept the reality of their lives. Importantly, they identified three core factors that helped them embrace their lived experiences as a positive caregiving dyad: filial piety, strengthened bonds and religious belief. The informants believed these factors contributed to their sense of control and legitimised their efforts and sacrifices throughout the caregiving journey. Filial piety was the main reason the informants eventually adapted to and accepted their additional role. All of them agreed that taking care of their parents was an act of devotion, and they never viewed caregiving as a burden despite the struggles. They also feared retribution if they refused to care for their parents, regardless of any prior conflict. Additionally, the informants shared that the beauty of caregiving was reflected in the bond between them and their care recipients. This bond grew stronger, and the once-strained relationships eventually healed. Most importantly, religious belief played a pivotal role in their adaptation and acceptance. The informants expressed that their reliance on God gave them strength and inner peace.

### Theme 4: Identity

The transition into a caregiving role often involves a shift in identity. Caregivers may struggle with changes in self-perception, particularly if caregiving becomes a predominant aspect of their lives. The informants perceived life as no longer belonging to them but to their care recipients. The overall experience of caregiving created an identity that distinctively described the family caregivers. All informants viewed caregiving as a source of personal fulfilment and a sense of purpose, as they provided essential support to their loved ones. However, it also involved personal sacrifices, affecting their emotional well-being and self-perception. They were expected to be empathetic, selfless and resilient. Emotional maturity also formed the foundation of their identity as family caregivers.

The themes revealed the difficulties and struggles of being a family caregiver, family issues and conflicts as major barriers to adjusting to changes, protective factors in adapting to and accepting the new norm and the development of the identity of family caregivers.

## Discussion

This study explored the lived experiences of Malaysian family caregivers of patients with chronic illnesses. The themes found were family caregivers’ adversities, adjustment challenges, adaptation and acceptance and identity. These themes depicted the phases and meaning of the caregivers’ experience, highlighting a transformation in their outlook on life. The existing literature supports the findings of this study. Understanding the challenges,^20,21^ a shift in priorities,^22,23^ efforts to overcome challenges^10,24-26^ and the formation of a new identity^27-29^ are factors associated with the meaning-making of life as a caregiver. Instead of focusing on the pain and stress of caregiving, caregivers relinquish the sense of meaning and lessons learnt, enabling them to fully support both themselves and their care recipients on their journey.

This study highlighted the essence of the cultural, emotional and spiritual dimensions of caregiving, particularly through filial piety and religious belief, which are key aspects in many Asian societies, including Malaysia. These findings are supported by recent studies^10,12,20^ showing that cultural values, religious beliefs, social backgrounds, family values and support, commitment, availability, practicality and patient needs shape the meaning and practicalities of caregiving. Family relationships are an important consideration in determining the right person to take over the caregiving role in the family, as they have a significant positive impact on family caregivers’ willingness to care.^30^ In the Malaysian family context, another study confirmed that cultural values, religious beliefs and family values inherited from older family members were also important in caring for aged parents or spouses.^31^ This is also supported by studies on Chinese families, in which filial piety was a crucial mediator in care experiences^32^ and a coping method across all Confucian cultures.^33^ Additionally, the present study found that strengthened bonds and religious belief were positive aspects of caregiving that shifted the family caregivers’ perspectives. While negative aspects of caregiving persisted, coping strategies and adaptation also helped the caregivers move beyond the difficult stages. Recent studies support the idea that when caregivers approach their role with a positive attitude, viewing it as meaningful and valuable, they not only form emotional connections with their care recipients but also promote personal growth and strengthen family bonds.^34,35^ This indicates that caregivers perceive their struggles and development as extending beyond mere structural challenges.

The presence of a chronic illness is a devastating stressor and negative experience for caregivers. Changes in family roles, routines and functioning may occur due to the detrimental effect the caregiving burden has on families.^22,23^ Family members are expected to adjust to function effectively, but not all are capable of achieving growth and establishing new perspectives. Some family caregivers are concerned about being the sole caregivers, and a third do not receive support from other family members.^36^ Family plays a crucial role in overcoming barriers, maintaining functionality and providing support in times of crisis. In the face of difficulty, family is key in the adjustment process, with the most resilient families adapting their coping strategies and recovering from crises. Therefore, understanding the resilience process of family adjustment due to chronic illnesses can contribute to developing comprehensive care strategies to promote better family adaptation and functioning. Family members need to be resourceful in many areas, such as emotional and social support, support groups, self-care management, financial assistance and information on treatment, nursing and caregiving methods.^10^ Inadequacy in any of these areas can hinder the adjustment process, as shown in the present study findings.

Protective factors in managing adversities and frustration are crucial. Spirituality, flexibility in responding to ongoing demands and emotional sharing are resilience processes that help families navigate periods of disintegration and vulnerability.^3,7^ Adaptation can be facilitated by increasing access to necessary information, providing financial and emotional support, identifying support resources and identifying and eliminating adaptation barriers such as restrictions in family relationships, blame, negative judgements from others, the chronic nature of the disease and limited or costly treatment options.^25^ Adjustment and adaptation require the reconstruction of family beliefs, development of new family organisation patterns, establishment and integration of social resources and improvement of communication and problem-solving.^26^ Hence, people’s beliefs, values and coping strategies related to religion and spirituality can serve as sources of hope, motivation and positivity.

In conclusion, this study highlights the impact of culture, religion and family cohesion on family caregivers’ lived experiences and caregiving trajectory. Constructive and positive family caregiving may be cultivated through increased knowledge in caregiving, greater appreciation and support from family members and acceptance of the new identity. This study provides healthcare professionals with approaches such as caregiver support programmes or workshops focused on emotional resilience or communication strategies to encourage family caregivers to maintain the well-being of their family members while caregiving and help them reach an optimum level of functioning as they learn to receive help and offer relief to each other. Future research is recommended to focus on family strengths, family functioning and practical family-based interventions that guide families in supporting caregivers and improving family relationships. Nonetheless, this study is limited to the lived experiences of Malay-Muslim family caregivers; therefore, family caregivers from different ethnic backgrounds may have different experiences. The impact on interfamily relationships among caregivers and family members could also be influenced by other confounding factors. It would be valuable to further explore ethnic differences in caregiving dyads.

## Data sharing statement

Raw data will not be shared to ensure the protection of participant confidentiality. Access to the data will be provided upon approval, with only the necessary information shared.

**How does this paper make a difference in general practice?**

- The findings provide insights into the perceptions and experiences of family caregivers regarding family caregiving throughout their caregiving trajectory.
- The findings reveal ways to cultivate a positive family caregiving experience and promote family support.
- The findings can guide healthcare professionals in implementing suitable and practical family-based interventions.
